# 

**Published:** 1874-05

**Authors:** 


THREE DOLLARS A YEAR. SPECIMEN COPIES, 30 CENTS.

VOL. XXXI. May, 1874. NUMBER 5.

THE

### Chicago Medical Journal.

J. ADAMS ALLEN, M.D., LL.D., WALTER HAY, M.D

EDITORS.

**TWELVE TIMES A YEAR.**

CHICAGO:

W. B. KEEN, COOKE & CO., PUBLISHERS,

NOS. 113 AND 115 STATE STREET.

